# Adrenalectomy promotes a permanent decrease of plasma corticoid levels and a transient increase of apoptosis and the expression of Transforming Growth Factor β1 (TGF-β1) in hippocampus: effect of a TGF-β1 oligo-antisense

**DOI:** 10.1186/1471-2202-7-40

**Published:** 2006-05-19

**Authors:** Javier A Bravo, Claudio S Parra, Sandor Arancibia, Sergio Andrés, Paola Morales, Mario Herrera-Marschitz, Luisa Herrera, Hernán E Lara, Jenny L Fiedler

**Affiliations:** 1 Department of Biochemistry and Molecular Biology. Laboratory of Neurobiochemistry. Faculty of Chemical and Pharmaceutical Sciences. Universidad de Chile, Chile; 2 Laboratory of Molecular Mechanisms of Neurodegenerative Diseases, Université de Montpellier, Montpellier, France; 3 Programmes of Molecular & Clinical Pharmacology ICBM, Faculty of Medicine, Universidad de Chile, Chile; 4 Human Genetics, ICBM, Faculty of Medicine, Universidad de Chile, Chile

## Abstract

**Background:**

Corticosterone reduction produced by adrenalectomy (ADX) induces apoptosis in dentate gyrus (DG) of the hippocampus, an effect related to an increase in the expression of the pro-apoptotic gene *bax*. However it has been reported that there is also an increase of the anti-apoptotic gene *bcl-2*, suggesting the promotion of a neuroprotective phenomenon, perhaps related to the expression of transforming growth factor β1 (TGF-β1). Thus, we have investigated whether TGF-β1 levels are induced by ADX, and whether apoptosis is increased by blocking the expression of TGF-β1 with an antisense oligonucleotide (ASO) administered intracerebrally in corticosterone depleted rats.

**Results:**

It was observed an increase of apoptosis in DG, 2 and 5 days after ADX, in agreement with a reduction of corticosterone levels. However, the effect of ADX on the number of apoptotic positive cells in DG was decreased 5 days after the lesion. In CA1–CA3 regions, the effect was only observed 2 days after ADX. TGF-β1 mRNA levels were increased 2 days after ADX. The sustained intracerebro-ventricular administration of a TGF-β1 ASO via an osmotic mini pump increased apoptosis levels in CA and DG regions 5 days after ADX as well as sham-operated control animals. No significant effect was observed following a scrambled-oligodeoxynucleotide treatment.

**Conclusion:**

The changes in both the pattern and the magnitude of apoptotic-cell morphology observed 2 and 5 days after ADX suggest that, as a consequence of the reduction of corticosteroids, some trophic mechanisms restricting cell death to a particular time window are elicited. Sustained intracerebral administration of TGF-β1 ASO increased the apoptosis promoted by ADX, suggesting that TGF-β1 plays an anti-apoptotic role *in vivo *in hippocampus.

## Background

Recent studies have proposed that cytokines and growth factors may influence the outcome of the damage induced by neurodegenerative diseases [1,2]. Transforming growth factor β1 (TGF-β1) represents the prototype of a large family of growth factors that regulate cell growth, development, differentiation and cell death [3,4].

TGF-βs have been detected at high concentrations in post-mortem brain from patients with Parkinson's [5] and Alzheimer's [6]diseases. Also, the presence of TGF-β1 promotes an accumulation of cellular mature amyliod protein precursor in a microglial cell line [7]. The expression of TGF-β1 is induced by hypoxia, ischemia and brain trauma in several brain regions, including the hippocampus [8-10]. However, whether the increased TGF-β1 expression observed in several neurological diseases has a beneficial or detrimental effect on neurons remains unclear.

Examples for both pro-apoptotic and neuroprotective roles of TGFβ1 have been described. *In vitro *studies have shown that immature cerebellar neurons exposed to TGF-β1 die by apoptosis [11]. Also, addition of TGF-β1 to organotypic cultures of postnatal mouse retina results in a prominent apoptosis [12,13]. In contrast, pharmacological administration of TGF-β1 prevents neuronal degeneration induced by excitotoxic injury *in vitro *[14] and rescues hippocampal CA1 neurons from post-ischemic cell death *in vivo *[15].

Targeted deletion of TGF-β1 in mice results in strain-dependent defects and embryonic lethality [16,17]. Although TGFβ1 knock-out mice in the NIH genetic background live for a few weeks after birth, they present increased numbers of apoptotic neurons in several brain regions including the neocortex, caudate putamen and cerebellum [18]. In addition, TGF-β1 deficiency in adult *Tgfβ1-/+ *mice results in increased neuronal susceptibility to excitotoxic injury in several structures including the hippocampus [18]. These observations have led to propose that TGF-β1 is a neuroprotective cytokine.

Although the mechanisms underlying the neuroprotective action of TGF-β1 have not been clarified, several reports have suggested that this cytokine may have a direct effect on apoptosis regulation. Administration of TGF-β1 to neuronal cultures prevents β-amyloid-induced apoptosis, probably by stimulating the expression of anti-apoptotic proteins, such as BCL-2 and BCL-X_L _[19]. In primary hippocampal neuronal cultures, it has been shown that TGF-β1 protects against the excitotoxicity induced by NMDA-dependent Ca^2+ ^conductance, probably via induction of BCL-2 gene expression [20]. In fact, some apoptotic signals promote mitochondrial membrane permeability, a process controlled by BCL-2[21], leading to cytochrome C release and pro-caspase-3 activation [22]. In agreement, it has been shown that TGF-β1 can prevent neuronal apoptosis induced by caspase-3 [23].

Several reports have demonstrated that adrenalectomy (ADX) induces apoptosis in the hippocampus [24-26], probably by depletion of corticosterone levels. Indeed, it has been found that ADX induces a strong decrease in plasma corticosterone levels and brain changes, including apoptosis and increased expression of TGF-β1 in hippocampus [27]. It has also been shown that ADX promotes apoptosis in granular cells of the dentate gyrus (DG), which can be prevented by corticosterone or aldosterone replacement [25]. The adrenalectomy-induced loss of negative feedback at the hypothalamic level, also increases the expression of corticotropin releasing hormone (CRH) in several limbic brain regions which could induce neuronal death [28]. However, intracerebroventricular infusion of ADX rats with (9–41)-alpha-helical CRH, a CRH antagonist, does not decrease the number of degenerating granule cells in the hippocampus [28] suggesting that the ADX-induced reduction in circulating levels of adrenal steroids, but not the rise in hypothalamic CRH release, is responsible for the apoptotic action on DG cells.

We have also reported that the ADX-induced apoptosis in the DG is associated with an increase in the expression of the pro-apoptotic gene *bax *[29] and activation of caspase-9 [30]. However, ADX also stimulated the expression of the anti-apoptotic gene *bcl-2 *[29]. Bye and collaborators have shown that TGF-β1 levels are increased following ADX, suggesting a neuroprotective effect for TGFβ1 [27].

Thus, we have investigated whether (i) TGF-β1 levels are induced by ADX, (ii) TGF-β1 has a neuroprotective role and (iii) intracerebral administration of an antisense oligonucleotide directed against TGF-β1 in rats depleted of corticosterone levels can block the TGF-β1-induced effects

## Results

### Adrenalectomy

To evaluate the effectiveness of ADX, serum corticosterone concentrations were measured by RIA in all animals. Corticosterone serum levels were significantly decreased after ADX (< 0.1 μg/dL; n = 20) compared to sham-operated controls (22 ± 2.5 μg/dL; n = 10). To decrease the chances of inducing any stress to the ADX- and sham-operated animals, special care was given to preserve the environmental conditions where the experimental animals were kept (temperature, light/dark cycle, humidity). The ADX animals also displayed a reduction in body weight gain respect to sham-operated animals, in agreement with a reduced corticosterone condition (data not shown).

### Apotosis induction in the hippocampus after adrenalectomy

**Figure 1:**
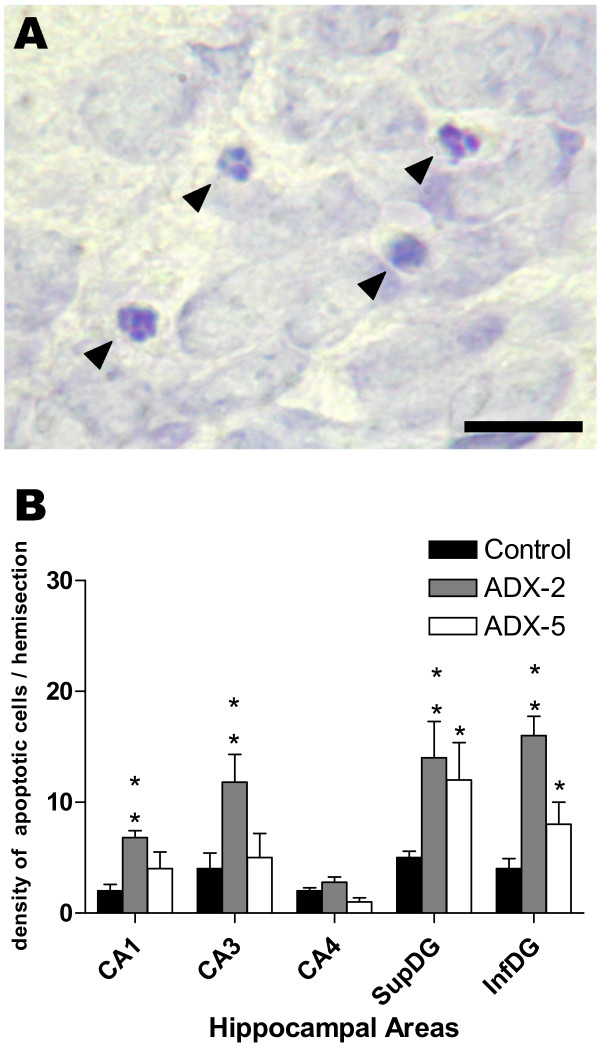
**Adrenalectomy (ADX) induced apoptosis in hippocampus**. Brain sections from 2-day (ADX-2) and 5-day (ADX-5) adrenalectomized animals were stained with 1% Cresyl Violet and the density of apoptotic cells in the left hippocampus was estimated. Apoptotic cells were counted in ten non-successive 14 μm thick sections at 100× magnification in Cornus Amonnis area (CA1, CA3, CA4) and supra- (SupDG) and infra- (InfDG) pyramidal layer of the dentate gyrus (DG). (A) Images illustrating the morphology of apoptotic nuclei (small condensed nuclei, extensive and tight condensation of nuclear material into darkly stained balls or disassembled pyknotic cells). (B) Bar graph represents the mean ± SEM (4–5 animals per group) of the density of apoptotic cells counted in each area for sham-operated animals (black columns), 2 days after ADX (dashed column) or 5 days after ADX (open column); the values obtained in 2-day and 5-day sham-operated animals were pooled because no differences were observed with time after the sham operation. The data was analysed using the non parametric Kruskal-Wallis test, followed by Dunns post-hoc test. * *p *< 0.05 and ** *p *< 0.01 compared to the corresponding sham-operated control. Scale bar = 20 μm.

As shown in Fig 1A, three morphological criteria were considered for assessing an apoptotic features: (i) pyknotic nucleus, (ii) nuclei with extensive and tight condensation of nuclear material into darkly stained balls, and/or (iii) segmented nuclei [26]. One of these profiles had to be fulfilled for counting as an apoptotic cell.

The density of cells showing apoptotic features was significantly increased in CA1–CA3 and in both supra- and infra-pyramidal layers of the dentate gyrus when comparing sections from 2-day ADX and sham-operated control animals (Fig. 1B). Apoptosis was still prominent in both blades of the DG 5 days after ADX (Fig. 1B). No significant changes in the density of apoptotic cells were observed in CA4 either 2 or 5 days after ADX.

### Adrenalectomy induces changes in TGFβ1 mRNA level in the hippocampus

**Figure 2:**
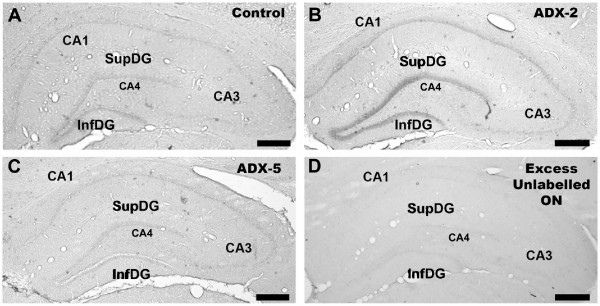
**ADX increased TGF-β1 NA expression in the hippocampus**. Photomicrographs show the changes in TGF-β1 mRNA levels in hippocampus of: (A) control (sham-operated), (B) 2 days after ADX (ADX-2), and (C) 5 days after ADX (ADX-5) animals. The *in situ *hybridisation was performed using a digoxigenin-labelled oligonucleotide probe complementary to TGF-β1 mRNA (524 pb – 563 bp; accession number NM_021578.1). The specificity of the hybridisation was evaluated using a 100-fold excess of unlabelled oligonucleotide (D). Scale bar = 500 μm.

TGFβ1 expression as assessed by *in situ *hybridisation was observed in different regions of the brain including neocortex and hypothalamus. The hybridisation signal was observed in the DG and cornus ammon fields CA1–CA3 of the hippocampus. Fig. 2 shows photomicrographs of the hippocampal regions after *in situ *hybridisation for TGF-β1 in brain sections from a sham-operated control animal (A), or a 2-day (B) or 5-day (C) ADX animals. The specificity of the hybridisation signal was evaluated with an excess of unlabelled probe (D).

**Figure 3:**
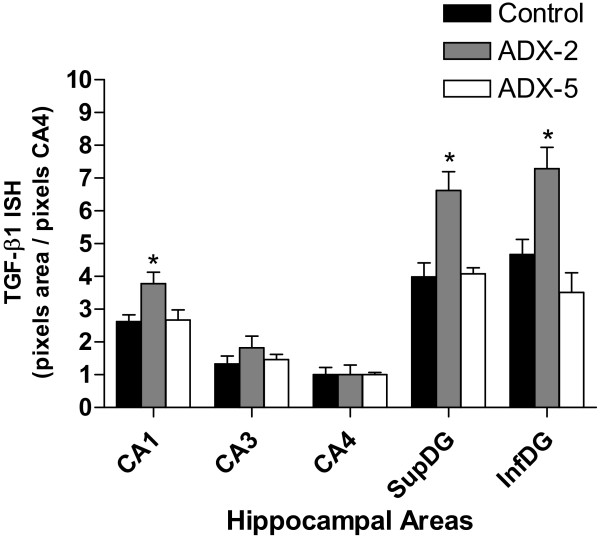
**TGF-β1 mRNA levels increased in CA1 and the dentate gyrus 2 days after ADX**. For semiquantitative analysis of TGF-β1 mRNA levels, densitometric measurements of each hippocampal subfield were performed using UN-SCAN-IT software (Silk Scientific Inc. Orem, UT, USA). The relative intensity of the hybridisation signals (pixels) in the different hippocampal regions was converted to a linear gray scale by assigning the highest optical density to the darkest staining and viceversa. The hybridisation signal in the *stratum radiatum *was considered as background and was subtracted from the optical density values obtained in the hippocampal cell layers. Bar graph represents the data expressed as optical density (pixels) normalized to the CA4 area. For each animal the value represents the average of measurements from 4–5 brain sections. Black bars, control animal; hatched bars, 2 days after ADX; open bars, 5 days after ADX. * *p *< 0.05 vs control

Sham-operated control animals showed TGF-β1 mRNA positive hybridisation in the neuropil of the supra- and infra-pyramidal layers of the DG (Fig. 2A). Expression of TGF-β1 was also detected in CA1, but the intensity of the hybridisation signal was weaker than in the DG. The CA3 and CA4 subfields showed the lowest intensity. Two days after ADX, an increase in the intensity of the TGF-β1 signal in the DG and CA1 was observed (Fig. 2B). However, 5 days after ADX the intensity of the hybridisation signal in the hippocampus was similar to that observed in sections from sham-operated animals (Fig. 2C). Also on the subgranular layer of DG there were some cells with positive TGF-β1 mRNA signal, but the method used to measure label intensity does not allow us to evaluate changes in the number positive cells (not shown). No hybridisation signal was observed when using an excess of unlabelled probe (Fig. 2D). The results obtained by *in situ *hybridisation were quantified and the intensity of the hybridisation signal was normalized to that of CA4, because the hybridisation signal did not show any change in that area under any of the experimental conditions tested (see Fig. 3).

Two days after ADX, the levels of TGF-β1 mRNA increased in CA1 by approximately 30% with respect to sham-operated animals (Fig. 3). Also, a significant increase in TGF-β1 mRNA was observed in supra- and infra-pyramidal layers of the DG (by 70% and 50%, respectively). In contrast, TGF-β1 mRNA levels in CA3 were not modified by ADX. TGF-β1 mRNA returned to sham-operated baseline levels 5 days after ADX in CA1 and the two blades of the DG.

### Lateral ventricle administration (i.c.v.) of an oligodeoxynucleotide antisense for TGF-β1 exacerbates hippocampal apoptosis induced by adrenalectomy

**Figure 4:**
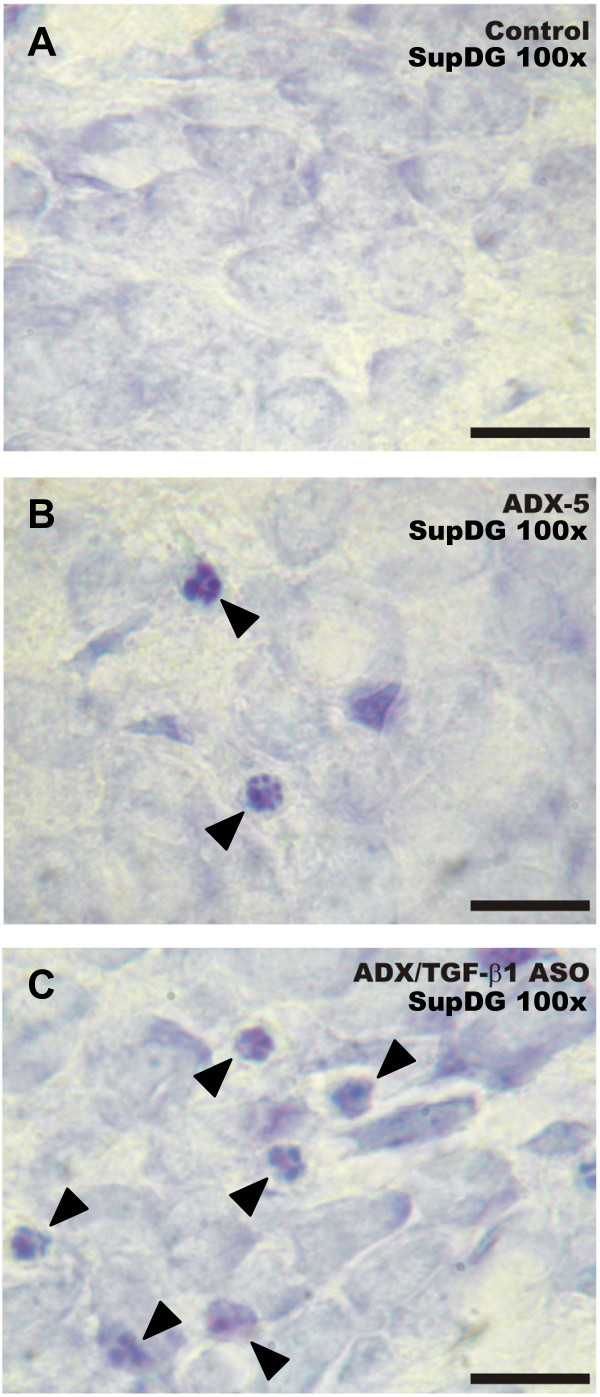
**Intracerebroventricular administration of antisense oligonucleotide directed against TGF-β1 increased the density of apoptotic cells in the dentate gyrus after five days of ADX**. The ASO, SCRO (10 μg/day) or vehicle were stereotaxically delivered into the lateral left ventricle, via a cannula connected to a subcutaneously implanted Alzet osmotic mini-pump, delivering the corresponding solution during 7 days. The animals were adrenalectomized, or sham-operated 2 days after the beginning of the i.c.v. treatment and euthanized 5 days after. Photomicrographs illustrate changes in apoptotic morphology in the suprapyramidal layer of the DG of: (A) control (saline-treated, sham-operated), (B) saline-treated, adrenalectomized (ADX-5), and (C) ASO TGF-β1-treated, adrenalectomized (ADX-5/TGF-β1 ASO) animals. Arrowheads denote pyknotic nuclei, nuclei containing clumped chromatin disassembling and degenerating nuclei. Scale bar = 20 μm.

To evaluate the role of TGF-β1 in the hippocampus under control and ADX conditions, an antisense oligonucleotide to TGF-β1 that has been successfully used by others [31] was infused into the lateral ventricle starting 2 days before ADX. TGF-β1 antisense produced an enhancement in the density of nuclei with apoptotic features in the DG 5 days after ADX (Fig. 4C) when compared to either sham-operated i.c.v. saline-infused (Fig. 4A) or to ADX i.c.v. saline-infused animals (Fig. 4B).

**Figure 5:**
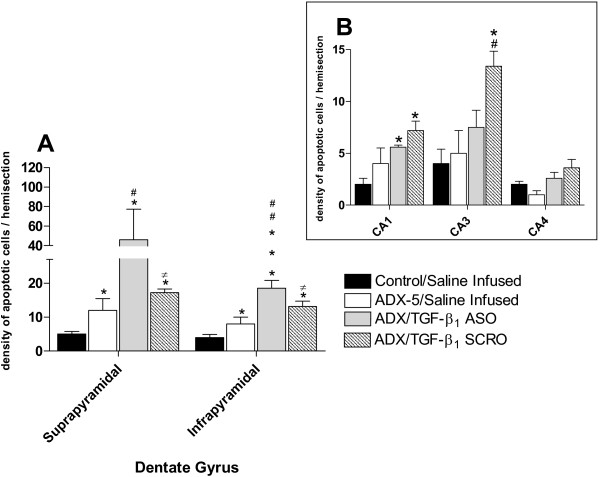
**Quantitative analysis of the effect of TGF-β1 ASO and SRO administration into the left lateral ventricle on hippocampal apoptotic morphology 5 days after adrenalectomy (ADX)**. Data represent the density of apoptotic cells (mean ± SEM) in the hippocampus ipsilateral to the side infused with the oligodeoxynucleotide or a saline solution. (**A**) Apoptosis observed in the suprapyramidal and infrapyramidal of dentate gyrus. Black columns, saline infused sham-operated animals; white columns, saline infused, ADX-5 animals; gray columns TGF-β1 ASO infused ADX-5 animals; dashed columns, TGF-β1 SCR infused ADX-5 animals. * *p *< 0.05 vs control; *** *p *< 0.01 vs control; # p < 0.05 vs ADX-5; ## p < 0.01 vs ADX-5, ≠ p < 0.05 vs ADX/TGF-β1 ASO. (**B**) Inset representing Cornus Amonis areas. Black columns, saline infused, sham-operated animals; white columns, saline infused, ADX-5 animals; gray columns TGF-β1 ASO infused ADX-5 animals; dashed columns, TGF-β1 SCRO infused ADX-5 animals. * *p *< 0.05 vs control; # p < 0.05 vs ADX-5.

Semiquantitative analysis demonstrated a >2-fold increase in the density of apoptotic cells in both blades of the DG, compared to that observed in sham- or ADX-operated animals infused with saline (Fig. 5A). This effect was specific because the administration of scrambled oligonucleotide (SCRO) did not have any effect compared to that seen in ADX animals treated with saline (Fig. 5A). Analysis of the results revealed that the density of apoptotic cells in the CA1 or CA4 regions was not statistically different between ASO- and SCRO- infused ADX rats (Fig. 5B). Nevertheless, administration of SCRO but not ASO to ADX animals induced a statistically significant increase in the density of apoptotic cells in the CA3 area compared to those in saline-infused rats (Fig. 5B). We also tried to correlate changes in TGF-β1 mRNA levels with TGF-β1 immunoreactivity in the hippocampus. However, the antibodies available did not produce positive immunoreaction neither in hippocampal slices nor control tissue (adrenal gland).

### Lateral ventricle administration (i.c.v.) of an antisense oligodeoxynucleotide against TGF-β1 promotes hippocampal apoptosis in control animals

**Figure 6:**
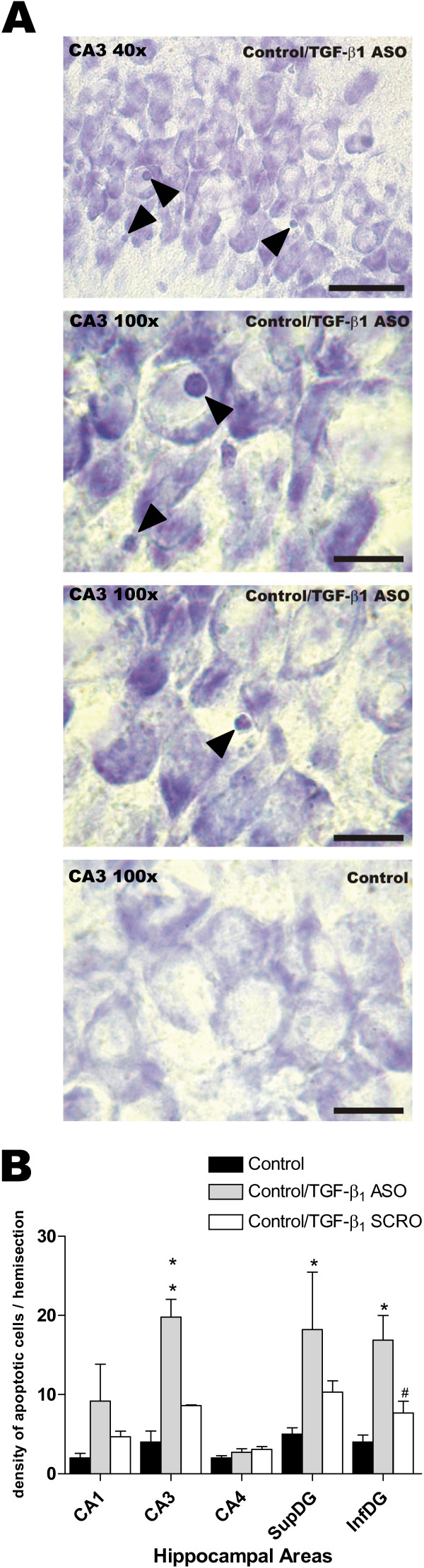
**TGF-β1 antisense oligonucleotide (ASO) administration into the left lateral ventricle induced hippocampal apoptotic morphology in a sham-operated control rat**. **(A) **Photomicrographs show the changes of apoptotic morphology in CA3 hippocampal subfield induced byTGF-β1 ASO i.c.v. treatment in sham-operated control animal. Upper microphotograph shows a 40× magnification (scale bar = 50 μm). The middle microphotographs show details of the same field at 100× magnification (scale bar = 20 μm). The lower microphotograph show CA3 area from a saline infused sham-operated animal. **(B) **Data represent the density of apoptotic cells (mean ± SEM from 4–5 animals) in the hippocampal areas ipsilateral to the side infused with saline (black bar), ASO (gray bar) or SCRO (open bar). * *p *< 0.05 vs control; ** p < 0.01 vs control; # p < 0.05 vs TGF-β1 ASO.

The effect of antisense oligonucleotide against TGF-β1 in sham-operated control animals in the CA3 region are illustrated in Fig. 6A. Compared to saline-infused controls, sections from sham-operated animals treated with ASO showed an increase in the number of apoptotic nuclei in the CA3 region whether the sections were observed at low (Fig. 6A, 40×) or high (Fig. 6A, 100×) magnification.

Semiquantitative analysis revealed a significant increase in the density of hippocampal cells undergoing apoptosis following ASO administration (Fig. 6B; p < 0.01 for the CA3 region and p < 0.05 for both blades of the DG when comparing with control untreated animals). No statistically significant effects were observed between control and SCRO-infused animals in any of the hippocampal areas studied.

## Discussion

The present study showed that ADX induced a strong decrease in plasma corticosterone levels, and increased TGFβ-1 expression and apoptosis in the hippocampus.

The decrease of corticosterone plasma levels by ADX should be accompanied by a decrease of aldosterone levels [25], and by an increase of hypothalamic secretion of CRH also accompanied by a raise in CRH expression in several limbic brain regions which could induce neuronal death [28]. The effect of ADX on apoptosis is probably due to corticosterone and aldosterone depletion, because the ADX-induction of apoptosis in granular cells of the DG is prevented by corticosterone or aldosterone replacement, but not by (9–41)-alpha-helical CRH, a CRH antagonist [28], indicating that the adrenal steroids exert a trophic influence on DG cells.

In agreement with a reduction in serum levels of corticosterone, apoptosis increased in the DG, 2 and 5 days after ADX. Also, a reduction (40%) in the density of apoptotic cells was observed in the infrapyramidal layer of DG 5 days after ADX, while corticosterone levels remained reduced. Several changes have been reported to occur in the DG after ADX. TUNEL positive cells in the hippocampus are more prevalent in the DG 5 days after ADX [26]. Moreover, induction of *bax *[29] and activation of caspases- 3 [32] and -9 [30] have been reported in the DG after short-term ADX. In contrast, the increase in density of apoptotic cells in the CA1–CA3 regions was only observed 2 days after ADX. The difference in both the temporal pattern and the magnitude of apoptotic-cell morphology observed 2 and 5 days after ADX in these hippocampal regions suggest that under these experimental conditions there are some trophic mechanisms restricting cell death to a particular time window after the insult provoked by the reduction of corticosteroids resulting from ADX. This restriction in cell death is probably related to the induction of the anti-apoptotic gene *bcl-2 *[29]. In agreement with this hypothesis, it has been reported that the removal of corticosteroids (3 days after ADX) triggers a specific gene expression profile in surviving dentate granule cells including the antiapoptotic *bcl-2 *gene, providing them the chance of survival and probably making them more resistant to the apoptosis observed after ADX [33].

Several types of brain injuries are able to induce the synthesis and/or the release of different growth factors (e.g., neurotrophins and cytokines) [1,2] providing neuroprotection [14,15]. TGF-β1, which is induced in the hippocampus after ADX [27] (and in this study), may also participate in the neuroprotective mechanisms. TGF-β1 is markedly increased in rat cerebral cortex and hippocampus after trauma, electrolytic lesion of the entorhinal cortex, or ischemia [8-10,34]. Moreover, the administration of TGF-β1 to neuronal cultures prevents the apoptosis induced by the β-amyloid through the induction of the antiapoptotic BCL-2 and BCL-X_L _proteins [19].

The majority of the studies performed to investigate the neuroprotective role of TGF-β1 are based on models where neuronal death is a continuum between necrosis and apoptosis [14,15], making it difficult to establish which one of these processes prevails. These processes are associated with different mechanisms buffering cell death. To differentiate which one of these processes is relevant, we used a model in which apoptosis takes place in a rather selective manner [24-26,29]. ADX has been proposed as a good *in vivo *model for hippocampal apoptosis, because it is a non invasive procedure to the brain and, as it is also shown in this study, it triggers apoptosis by corticosteroid and aldosterone reduction only [24-26]. Thus, we have evaluated with the ADX model if the expression of TGF-β1 is modified.

Primary rat hippocampal neuronal culture secretes TGF-β1 making feasible an autocrine and/or paracrine role in the hippocampus [35]. TGF-β1 mRNA and its protein have been localised in both hippocampal neurons and glia [32,36]. Hippocampal CA1–CA4 neurons display immunoreactivity for active TGF-β1 under physiological conditions, although mRNA expression was low [36,37]. These evidences suggest basal expression of TGF-β1 in the hippocampus. In the present study, expression of TGF-β1 mRNA was observed in the neuropil of the supra and infra-pyramidal layers of DG and comparatively weaker in the CA1 and CA3 subfields. Taken together, these results indicate the existence of basal expression of TGF-β1 in the hippocampus.

Labelling for TGF-β1 mRNA over OX-42-positive microglia with an activated morphology in the dentate gyrus granule cell layer has been observed 3 days after ADX [32]. In our studies, the expression of TGF-β1 mRNA showed a transient increase in both CA1 and supra- and infrapyramidal-layers of the DG 2 days after ADX with values returning to control levels 5 days after ADX. The plastic changes observed in the present study could be related to the permanent reduction of corticosteroid plasma levels and/or to cell death promoted by ADX.

It is well established that corticosteroids regulate many physiological processes through their binding to glucocorticoid receptors (GR). The ligand-activated receptor dimer activates gene expression by binding to specific DNA sequences (**g**lucocorticoid **r**esponse **e**lement, **GRE**) in the promoter regions of glucocorticoid-regulated genes [38]. In contrast to the regulation of these classical GREs, the repression of negatively regulated target genes is mediated by trans-repression and/or by the binding of the GR to negative GREs (nGREs) [38]. It is possible that nGREs are also present in the TGF-β1 gene promoter region. The corticosterone reduction switches off the action of the nGREs, enhancing the expression of TGF-β1. At least in humans, it is known that human TGF-β1 gene has a GRE in its promoter [39], probably acting as a repressor of the TGF-β1 gene expression. Indeed, the increase in the expression of hippocampal TGF-β1 by ADX is prevented by corticosterone administration [40]. However, this mechanism cannot totally account for the normalization of TGF-β1 mRNA levels observed 5 days after ADX in our experiments.

During the early stages of apoptosis, phosphatidylserine is exposed to the cell surface, triggering cell engulfment by neighboring cells or phagocytes. However, exposure of phosphatidyl-serine to the outer face of plasma membrane in the apoptotic cells acts as a stimulus to induce the expression of TGF-β1 by microglia [41]. Therefore, the up-regulation of TGF-β1 observed following ADX may result from neuronal death itself, similar to that reported by other scientists [8-10,42]. However, other authors have observed an increase in TGF-β1 expression 7 days after ADX although in different rat strains [40]. Recently, the same authors showed the time course of TGF-β1 mRNA expression in the DG which is increased 2 days after ADX (similar to our results) reaching maximum 3 days after ADX [32]. Also the levels of expression are mantained 7 days after ADX, a period where the apoptosis is still occurring. However these authors did not study the TGF-β1 expression after 5 or more days after ADX. More recently, Battista et al [43] showed an increase in TGF-β1 by RT-PCR in the hippocampus 3 days after ADX. It is possible that the ADX-induced expression of TGF-β1 could be biphasic, i.e. an early generalized induction of TGF-β1 mRNA in the hippocampus (CA and DG areas) which is maintained for a few days after ADX (1–3 days) and thereafter the gene is re-expressed between 5–7 days of ADX and persists over ADX period in areas undergoing neuronal death, i.e. in the DG. A similar pattern of TGF-β1 induction has been reported in the post-ischemic adult rat hippocampus [8,36].

Thus, it was interesting to study the effect of blocking TGF-β1 mRNA translation by an antisense oligonucleotide. We used this approach instead of blocking the TGF-β1 peptide using neutralizing antibodies, because the oligonucleotide is more diffusible that an antibody. Also the administration of a neutralizing antibody requires a single application near to the site of interest, i.e. close to the DG or CA areas, making it feasible to induce tissue damage and by this means microglia activation. We used a TGF-β1 ASO developed by Le Roy et al. [31], who demonstrated that antisense prevented the translation of TGF-β1 mRNA by Leydig cells and, consequently, reduced TGF-β1 immunoreactivity, which was not observed following administration of a corresponding SCRO

In this study, sustained i.c.v. administration of that TGF-β1 ASO via an osmotic mini pump increased apoptosis levels in CA and DG regions 5 days after ADX, but also in sham-operated control animals. Indeed, when TGF-β1 ASO was administered to sham-operated animals, apoptotic cells were increased in both CA3 and DG regions, while in ADX-animals the effect was restricted to the DG. The CA3 area shows low levels of TGF-β1 mRNA, both in control and ADX animals, indicating that its expression is independent of corticosterone levels. The finding that ASO administration promotes apoptosis in CA3 led us to propose that low levels of TGF-β1 are enough to protect cells against apoptosis in this hippocampal subfield. Also TGF-β1 is capable of suppressing the levels of TNF-α [44], suggesting that the reduction of TGF-β1 by ASO treatment might induce the expression of this pro-inflammatory cytokine, that can also promote apoptosis through its receptor[45]. It is remarkable that CA3 field is normally resistant to ischemia [46] and ADX (5 days, Fig. 1) and this phenomena could be achieved through a tonic expression of TGF-β1 [36]. Interestingly TGF-β1 deficiency in adult Tgfβ1-/+ mice results in increased neuronal susceptibility to excitotoxic injury in several structures including the hippocampus [18].

The lack of effect of the TGF-β1 ASO on ADX CA3 may be explained by other neurotrophic factors elicited by the lesion. In fact, it has been shown that ADX promotes the induction of BDNF in pyramidal regions of the hippocampus [47,48].

In the present study, SCRO administration did not induce any effect on apoptosis levels, except in CA3 of ADX animals, where there was an increase of apoptosis levels following intracerebral SCRO administration. While we do not have any explanation for this effect, it may be speculated that is probably due to the presence of an unmethylated CpG dinucleotide in the SCRO sequence. This a motif that has been reported to stimulate microglia and astrocytes in the CNS, releasing TNF-α, a pro-inflamatory cytokine[49]. This cytokine may exert in turn neuronal injury in vulnerable regions [50], under conditions where there is a decrease of corticosterone levels, like following ADX.

## Conclusion

ADX promotes a permanent decrease of plasma corticoid levels and transient increase of apoptosis and TGF-β1 expression in hippocampus, reaching a maximum 2 days after ADX. The effect was mainly observed in supra- and infra-layers of the DG, where the expression of TGF-β1 mRNA was stronger than in any other regions of the hippocampus.

Administration of a TGF-β1 ASO promoted hippocampal apoptosis, both in sham- and ADX-animals. In sham-operated animals, apoptosis was increased in both CA3 and DG regions, while in ADX-animals the effect was restricted to the DG. The lack of effect of the TGF-β1 ASO on ADX CA3 can be explained by other neurotrophic factors elicited by the lesion.

These observations, together with previously published reports indicating an increase in the expression of TGF-β1 in response to brain injuries, suggest that TGF-β1 exerts neuroprotection in adult nervous system.

## Methods

### Animals

Adult (200–220 g) male Sprague-Dawley rats were used in all experiments. The animals were kept under temperature (22°C–24°C), humidity (50%) and 12 h regulated light-dark cycle conditions, with food and water *ad libitum*. All procedures and care of the animals were conducted following protocols approved by the local Institutional Ethical Committee of the Faculty of Chemical and Pharmaceutical Sciences, University of Chile, in compliance with guidelines published in the National Institutes of Health *Guide for the care and use of laboratory animals*.

### Design and synthesis of oligodeoxynucleotides

All phosphorothioates oligodeoxynucleotides were purchased in purified form from Integrated DNA technologies (INC, Coralville, IA, USA), single-end capped (sulphur modification on the phosphate group between the first and last base pairs), displaying low toxicity for the central nervous system [51,52]. Oligodeoxynucleotides were reconstituted in ultra-filtered saline at a concentration of 417 pg/μL. The antisense to TGF-β1 was a 15 base oligomer that had been previously used in its full phosphorothioate form [31]. Its sequence was 5'-G-_S_-GAGGGCGGCATGG-_S_-G-3' (the -_S_- denotes locations of the sulphur modification) and complementary to the translation-initiation region of the rat TGF-β1 mRNA (410–424 pb of rat TGF-β1 cDNA; Genbank accession number NM_0215781). A scrambled oligodeoxynucleotide (SCRO), which had a randomly ordered but identical base content to the antisense was used as a control. Its sequence was 5'-A-_S_-GGTGGGAGGCGGC-_S_-G-3'. All oligodeoxynucleotides used in this study were subjected to a BLASTN search on the National Center for Biotechnology Information BLAST server using the Genbank database. The antisense oligodeoxynucleotide sequence had a 100% positive match for its mRNA sequence. The SCRO produced no positive matches.

### Surgery

#### Adrenalectomy (ADX)

Animals were anaesthetised with isofluorane (1.5% v/v air) and subjected to a bilateral ADX. The animals were kept with a 0.9% NaCl drinking solution to compensate the sodium deficit induced by the ADX. The sham-operated rats underwent a skin and lumbar muscle incision, leaving the adrenal glands untouched.

#### Oligodeoxynucleotide-i.c.v. administration

The rats were anaesthetised as above and fixed in a David Kopt stereotaxic frame. Guide cannulae were implanted into the left lateral ventricle (i.c.v., intra cerebro-ventricular infusion) (Bregma -0.8 mm, lateral 1.5 with a depth of 3.7 mm below the dura) according to Paxinos and Watson [53]. The cannula was then connected by a catheter to an ALZET^® ^mini-osmotic pump (Model 2001; Palo Alto, CA) implanted subcutaneously in the scapular region delivering at a rate of 10 μg oligodeoxynucleotide/day. For saline-treated control animals, the ALZET pump was filled with sterile saline solution. After 2 days of oligodeoxynucleotide infusion, the rats were subjected to either ADX or a sham operation as described above and maintained for further 5 days after surgery.

#### Tissue fixation

Two or five days after ADX, the animals were anesthetized by intramuscular injection of xylazine (5 mg/kg) and ketamine (50 mg/kg). A blood sample was taken from the heart and the obtained serum was kept at -20°C for corticosterone determination. Animals were then transcardially perfused with 100 mL of 0.9% NaCl followed by 500 mL of 4% paraformaldehyde in 0.1 M phosphate buffer (PB) pH 7.4. The brain was dissected from the skull, post-fixed in the same buffer overnight and then immersed in 30% sucrose in 0.1 M phosphate buffered saline (PBS) at 4°C for 2 days. Coronal sections (14 μm thick) were sliced from the frozen fixed brains and processed for Nissl staining.

#### Apoptotic morphology

Sections were dipped in 1% Cresyl Violet for 20 min, dehydrated in alcohol and mounted in Entellan (Merck, Darmstadt, Germany). Apoptotic nuclei were identified by using the criteria proposed by Greiner et al [26]. In short, one of the following criteria had to be fulfilled to count for an apoptotic cell: (i) pyknotic nucleus; (ii) extensive and tight condensation of nuclear material into darkly stained balls, and/or (iii) segmented nuclei [26]. The density of apoptotic cells per hemisection was calculated from ten non-successive coronal sections inspected at 100× for each animal [29], and expressed as the means ± SEM of 4–5 animals.

### In situ hybridisation for TGFβ1 mRNA

The *in situ *hybridisation was conducted with an oligodeoxynucleotide probe complementary to TGF-β1 mRNA (524–563 pb access number NM_021578.1) labelled with a digoxigenin oligonucleotide 3'-OH tailing kit (Roche, Molecular Biochemicals, Mannheim, Germany). The hybridisation was conducted as previously described [29]. The specificity of the hybridisation was evaluated by the use of 100 fold excess of the unlabelled oligodeoxynucleotide. For semiquantitative analysis, densitometric measurements of each hippocampal subfield were analysed using UN-SCAN-IT software (Silk Scientific Inc. Orem, UT, USA). A grey scale was used for measuring the intensity of the signals (pixels) observed in the hippocampal regions, i.e. the darkest staining the highest optical density; the lightest staining the lowest optical density. The hybridisation signal in the stratum radiatum was considered as background and was subtracted from the optical density values obtained in the hippocampal cell layers. For each animal the value represents the average from 4–5 brain sections. In order to normalize the different experimental conditions, all the data are expressed as optical density respect to the CA4, because the intensity in that region did not change following any of the experimental conditions.

### Corticosterone radio immunoassay

Corticosterone levels were determined by RIA according to the instruction of the manufacturer (Sigma, St. Louis, MO, USA), as described previously [26]. In short, the rat serum was diluted 1:10 in 0.05 M Tris-HCl, pH 8.0, containing 0.1 M NaCl, 0.1% BSA and then warmed at 50°C for 30 min. The anti-corticosterone antibody (Sigma-Aldrich, St Louis MO, USA; rabbit) was added to 0.1 ml sample or to 0.1 ml of the corticosterone standard (31–1000 pg). After 30 min of incubation, a ^3^H-corticosterone tracer (specific activity 100 Ci/mmol, Sigma-Aldrich, St Louis MO, USA) was added to each tube and incubated for 1 hour at 37°C. Finally, 0.2 ml cold dextran coated charcoal was added to the standard and/or samples, centrifuged at 2000 × g for 15 minutes at 4°C. The radioactivity was measured in the supernatant. The quantity of corticosterone in serum samples was estimated using the Prism software 4.0 (GraphPad Software, San Diego, CA). Intra and inter assays variation was less than 5%. The minimal detectable level of corticosterone was 0.1 μg/assay.

### Statistical analysis

Descriptive statistic for individual parameters are reported as mean ± SEM. The Prism software 4.0 (GraphPad Software, San Diego, CA) was used for statistical analysis and graph representation. Treatment effect was assayed with the non parametric Kruskal-Wallis test, followed by Dunns post-hoc test. Differences were considered to be significant when the probability of their occurrence as a result of chance alone was less than 5%.

## Abbreviations

ADX, Adrenalectomy

ASO, anti-sense oligodeoxynucleotide

CRH, corticotropin releasing hormone

DG, dentate gyrus

i.c.v., intra-cerebro ventricular

SCRO, scrambled oligodeoxynucleotide

TGFβ1, Transforming Growth Factor beta -1

s.c, sub-cutaneous

## Authors' contributions

BJA carried out experimental design, sample preparation, *in situ *hybridisation experiments, statistical analysis, interpretation of data and involved in drafting the manuscript. PCS participated in its design, surgery, data analysis and helped draft the manuscript. AS was involved in drafting the article critically. AS, MP, HL, LHE were involved in the critical review of the manuscript. H-MM was involved in revising the manuscript critically. FJL conceived, designed and coordinated the study, analysed, interpreted and drafted the manuscript.
